# Comparison of the clinical characteristics and clinical outcomes of culture-positive septic shock and culture-negative septic shock among pediatric patients

**DOI:** 10.1371/journal.pone.0288615

**Published:** 2023-07-14

**Authors:** Da Hyun Kim, Seong Jong Park, Won Kyoung Jhang

**Affiliations:** Division of Pediatric Critical Care Medicine, Department of Pediatrics, Asan Medical Center Children’s Hospital, University of Ulsan College of Medicine, Seoul, Korea; Kaohsuing Medical University Hospital, TAIWAN

## Abstract

**Objectives:**

Among pediatric patients with septic shock, culture-negative septic shock (CNSS) is common but there have been limited data on its clinical characteristics and prognosis. We compared the clinical characteristics and clinical outcomes between culture-positive septic shock (CPSS) and CNSS in pediatric patients.

**Design:**

Retrospective single-center study.

**Setting:**

Pediatric intensive care unit (PICU) of a tertiary referral hospital.

**Patients:**

All pediatric patients who were admitted to the PICU due to septic shock between January 2010 and November 2021, except for those with fungal or viral infections and those who expired on the day of admittance to the PICU. The primary outcome was 30-day mortality and in-hospital mortality.

**Interventions:**

None.

**Measurements and main results:**

A total of 294 patients (CPSS group, n = 185 [62.9%]; CNSS group, n = 109 [37.1%]) were included. The rates of 30-day mortality and in-hospital mortality (30-day mortality 22.7% vs 22%, in-hospital mortality 29.7% vs 25.7%) were not significantly different between the CPSS group and the CNSS group. The two groups showed comparable results in clinical outcomes such as the requirement for mechanical ventilator and renal replacement therapy, PICU stay duration, and the duration of MV and vasopressor/inotrope support. Among the CPSS group, 98 (53%) patients who were infected with multi-drug resistance (MDR) bacteria had significantly higher rates of 30-day mortality and in-hospital mortality than those infected with non-MDR bacteria.

**Conclusions:**

Among pediatric patients, the CPSS group and CNSS group did not show significant differences in clinical features and mortality. Among the CPSS group, those with MDR bacteria had poorer prognosis.

## Introduction

During the past decades, the incidence of septic shock has been increasing worldwide [1]. Mortality of septic shock is high and ranges from 20% to 40% according to the severity of the illness [2]. Bacteria are the most common cause of sepsis [3], and previous studies showed that identification of the pathogen provides vital information for choosing the appropriate antibiotics, which leads to better clinical results [4–6].

Among septic shock patients, there are some whose pathogen is not identified in culture, which is referred to as culture-negative septic shock (CNSS). Although the proportion of patients with CNSS among total septic shock patients is reported to be as high as 30% to 50%, the clinical characteristics of CNSS are not well-known [7, 8]. Yet, there could be crucial differences between culture-positive septic shock (CPSS) and CNSS, such as pathogenesis, epidemiology, and effectiveness of treatment [9]. Although physicians have studied the clinical outcomes between patients with CPSS and those with CNSS, this subject still requires further investigation [10–15].

While many studies compared the clinical features between CPSS and CNSS in adult patients, only few studies directly compared the two groups in the pediatric population [16–18]. In addition, the relationship between clinical characteristics and proven bacteria is not well-studied in pediatric patients with septic shock. Therefore, this study aimed to investigate the clinical features between patients with CPSS and those with CNSS among pediatric patients, and analyze the clinical features according to proven bacteria.

## Materials and method

### Study design

This was a single-center retrospective observational study. The medical records of all critically ill pediatric patients admitted to the 14-bed multidisciplinary pediatric intensive care unit (PICU) of Asan Medical Center, a tertiary academic referral hospital in Seoul, South Korea, between January 2010 and November 2021 were screened. Among them, we included patients with septic shock, which was defined as a clinical construct of sepsis with cardiovascular dysfunction despite adequate volume resuscitation [19]. We excluded patients with fungal or viral infection and those who expired on the day of a admittance to the PICU.

We divided the patients into two groups according to the culture result (i.e., CPSS vs. CNSS). Also, to investigate the mortality according to the type of bacteria (presence of multi-drug resistance [MDR] bacteria), we divided the CPSS group into the MDR group and non-MDR group. MDR was defined as isolates being non-susceptible to at least 1 agent in 3 or more antimicrobial categories listed in the standard definitions for acquired resistance [20]. All medical staff were trained to follow the Surviving Sepsis Campaign guidelines in the event of septic shock [19]. Therefore, all patients with septic shock should be done culture before administrating antibiotics. This retrospective study was approved by the institutional review board committee of Asan Medical Center (study title: clinical outcomes between culture positive and culture negative pediatric septic shock, IRB approval number: 2022–1128). It was waived the need for parental consent by Asan Medical Center Institutional Review Board Scientific Review Committee and it was determined to be exempt from regulatory oversight.

### Data collection

We collected the following data by reviewing the electronic medical records of all included patients: patient demographics, microbiological information of culture results, underlying disease, initial vital signs, duration of PICU stay, neurologic outcome, laboratory findings (e.g., complete blood cell count, chemistry, lactate, C-reactive protein, and arterial blood gas analysis). To evaluate the severity of organ dysfunction, we calculated the Pediatric risk of mortality (PRISM) III and pediatric sequential organ failure assessment (pSOFA) scores using the worst value of each parameter within the first 24 hours of admission to the PICU [21, 22].

Septic shock is a disease that can increase mortality in a short period of time and can affect mortality in the long period for reasons such as multi-organ failure. Therefore, in order to confirm the clinical difference between these two groups, 30-day mortality and in-hospital mortality were set as primary outcomes. The secondary outcomes were the requirement of mechanical ventilator (MV) and renal replacement therapy (RRT) and the duration of MV and vasopressor/inotrope support.

### Statistical analysis

All data were analyzed using IBM SPSS Statistics for Windows, version 21.0 (IBM Corp, Armonk, NY, USA). Categorical variables are expressed as numbers and percentages and analyzed using the chi-squared test or two-tailed Fisher’s exact test as appropriate. Continuous data are presented as mean ± standard deviation (SD) or median with interquartile range (IQR) and compared using two-tailed Student’s *t*-test. Kaplan–Meier survival curves with the log-rank test were stratified according to culture results and the presence of MDR bacteria. *P*-values lower than 0.05 were considered statistically significant.

## Results

This study included 294 pediatric patients (CPSS group, n = 185 [62.9%]; CNSS group, n = 109 [37.1%]) with septic shock who were admitted to the PICU. **Table 1** shows the baseline characteristics of the patients. The mean age was 91.9 ± 73.6 months and the body weight was 24.8 ± 20.3 kg. Culture was performed on all patients suspected of sepsis. The most common culture specimen was blood (n = 289 [98.3%]), followed by sputum (n = 233 [79.3%]) and urine (n = 165 [56.1%]). The rate of blood culture positivity was 83.4% and the rate of ascites positivity was 73.7% (**S1 Table**).

**Table 1:** Baseline characteristics of the study population.

	Total (n = 294)	CPSS (n = 185)	CNSS (n = 109)	*p*-value
Age (months)	82.4 (18.3–167.6)	56.4 (15.5–153.8)	120.1 (40.5–180.1)	<0.001
Male	172 (58.5)	115 (62.1)	57 (52.3)	0.11
Body weight (kg)	24.8 ± 20.3	21.6 ± 18.1	30 ± 22.6	<0.01
Underlying disease				0.25
Hemato-oncologic disease	96 (32.7)	65 (35.1)	31 (28.4)	
Cardiac disease	62 (21.1)	42 (22.7)	20 (18.3)	
Pulmonary disease	52 (17.7)	33 (17.8)	19 (17.4)	
Neurologic disease	28 (9.5)	17 (9.2)	11 (10.1)	
Renal disease	22 (7.5)	9 (4.9)	13 (11.9)	
Liver disease	13 (4.4)	8 (4.3)	5 (4.6)	
Gastrointestinal disease	8 (2.7)	4 (2.2)	4 (3.7)	
Metabolic disease	7 (2.4)	4 (2.2)	3 (2.8)	
No disease	6 (2)	3 (1.6)	3 (2.8)	
Initial vital signs				
Systolic blood pressure (mmHg)	75.5 ± 16.1	73.8 ± 15.6	78.3 ± 16.6	0.022
Diastolic blood pressure (mmHg)	41.8 ± 11.8	40.5 ± 11	44.2 ± 12.7	0.012
Heart rate (/min)	147.8 ± 30.2	150 ± 33.2	143.9 ± 24.1	0.09
Body temperature (°C)	37.8 ± 1.4	37.9 ± 1.3	37.5 ± 1.5	0.035
Laboratory				
WBC (×10^3^/μL)	12.6 ± 14.8	11.8 ± 13.4	13.9 ± 17.1	0.29
Hb (g/dL)	9.0 ± 2.1	8.8 ± 1.9	9.3 ± 2.3	0.07
PLT (×10^3^/μL)	126.7 ± 211.3	124.6 ± 248.3	130.2 ± 126.7	0.80
PT (INR)	2 ± 2	2.1 ± 2.2	1.9 ± 1.8	0.38
Lactate (mmol/L)	4.7 ± 4.4	5 ± 4.5	4.2 ± 4.1	0.08
BUN (mg/dL)	29.5 ± 25.3	27.5 ± 21	32.8 ± 31.1	0.08
Creatinine (mg/dL)	1.3 ± 1.7	1.1 ± 1.5	1.7 ± 1.9	0.001
Total bilirubin (mg/dL)	2.6 ± 5.3	2.6 ± 5.5	2.5 ± 5	0.87
CRP (mg/dL)	16.3 ± 10.9	16.1 ± 10.9	16.5 ± 11	0.76
Albumin (g/dL)	2.8 ± 1.8	2.7 ± 0.6	2.9 ± 2.8	0.45
Score				
PRISMIII	13.4±8	13.4 ± 7.8	13.4 ± 8.3	0.96
pSOFA	9.5±3.9	9.7 ± 4	9.3 ± 3.7	0.45

Data are presented as median (interquartile range), n (%), or mean ± standard deviation

Abbreviations: CPSS = culture-positive septic shock; CNSS = culture-negative septic shock; WBC = white blood cell; Hb = hemoglobin; PLT = platelet; PT = prothrombin time; INR = international normalized ratio; BUN = blood urea nitrogen; CRP = C-reactive protein; PRISM = pediatric risk of mortality; pSOFA = pediatric sequential organ failure assessment.

When the patients were analyzed according to the culture result, those with CPSS were younger (56.4 vs. 120.1 days, *p* < 0.001) than those with CNSS. Time from recognition of sepsis to antibiotic administration was not significantly different between the CPSS group (40.5 ± 73.5 min) and the CNSS group (45.7 ± 98.1 min; *p* = 0.63). On average, both groups were administered antibiotics within an hour.

**Fig 1:**
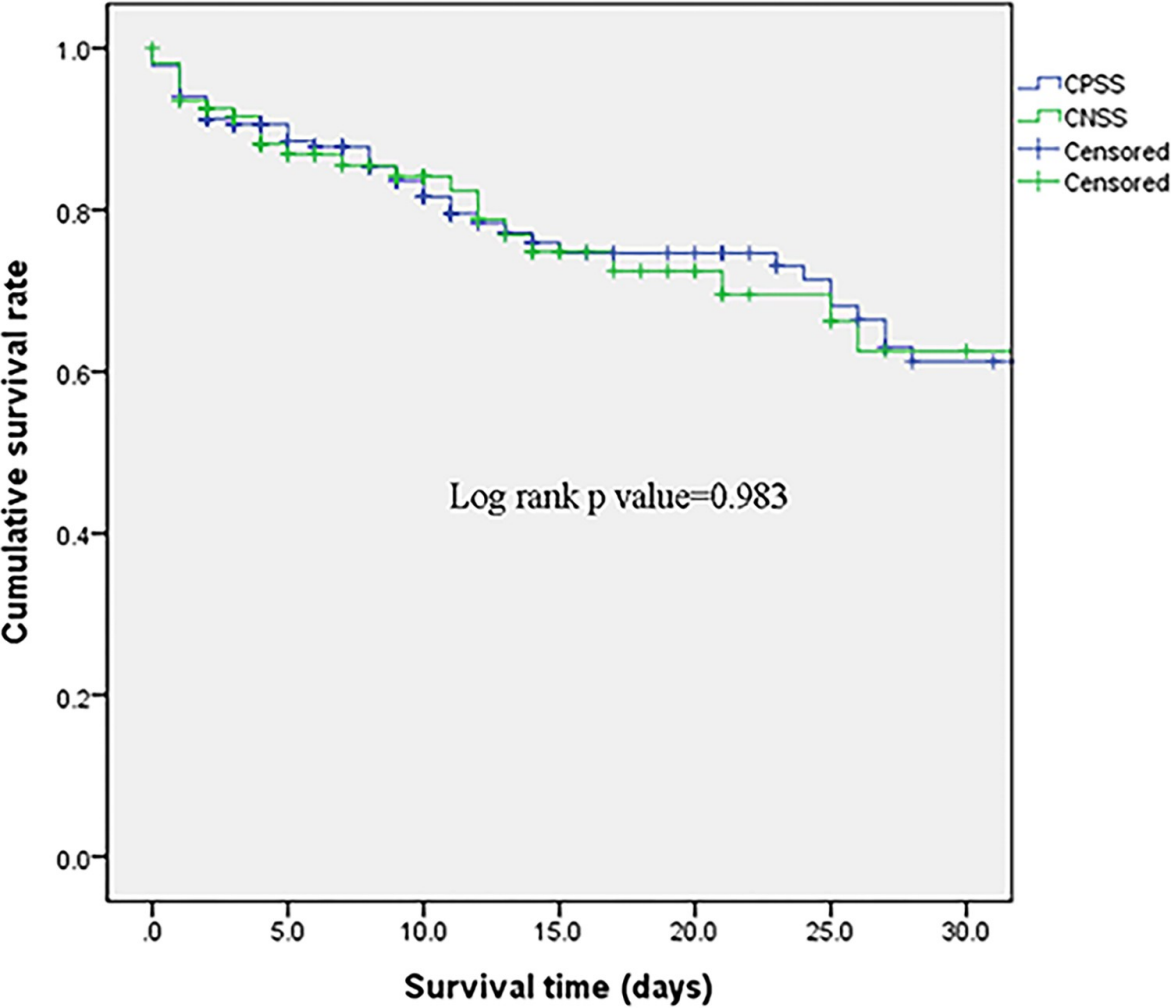
Kaplan–Meier survival curves according to the culture result.

As for the clinical outcomes, the two groups had similar mortality rates in terms of both 30-day mortality (22.7% vs. 22.0%; *p* > 0.99, log-rank *p* = 0.98) and in-hospital mortality (29.7% vs. 25.7%; *p* = 0.50)) (**Table 2**, **Fig 1**). In addition, there were no significant differences in the requirement of MV and RRT. The two groups had similar duration of PICU stay, duration of vasopressor/inotrope support, and duration of MV.

**Table 2:** Clinical outcomes according to the culture result.

	CPSS (n = 185)	CNSS (n = 109)	*p*-value
30-day mortality	42 (22.7)	24 (22.0)	> 0.99
In hospital mortality	55 (29.7)	28 (25.7)	0.50
Duration of PICU stay (days)	18.3 ± 30.7	16 ± 24.3	0.48
Duration of hospital stay (days)	76.5±104.2	67.8±119.5	0.53
MV requirement	93 (50.3)	49 (45)	0.40
RRT requirement	35 (18.9)	23 (21.1)	0.65
Time from recognition of sepsis to antibiotics administration (mins)	40.5 ± 73.5	45.7 ± 98.1	0.63
Duration of vasopressor/inotrope infusion (days)	6.8 ± 7	6 ± 7.3	0.32
Duration of MV (days)	10.4 ± 18.3	8.1 ± 14.2	0.23

Data are n (%) or means ±standard deviation

Abbreviations: CPSS = culture-positive septic shock; CNSS = culture-negative septic shock; PICU = pediatric intensive care unit; MV = mechanical ventilator; RRT = renal replacement therapy

In the CPSS group, *Staphylococcus* species were the most common bacteria (25.9%), followed by *Pseudomonas* species (14.6%) and *Klebsiella* species (14.6%). Overall, gram-negative species (55.2%) were more common than gram-positive species (44.8%). *Staphylococcus* species infection was the most common infection among those with 30-day mortality (16.7%). Patients with *Acinetobacter baumanii* infection had the highest 30-day mortality (60%), followed by *Enterobacter* species infection (56%) and *Burkhoderia cepacia* infection (33%) (**S1 Fig, S2 Table**).

**Fig 2:**
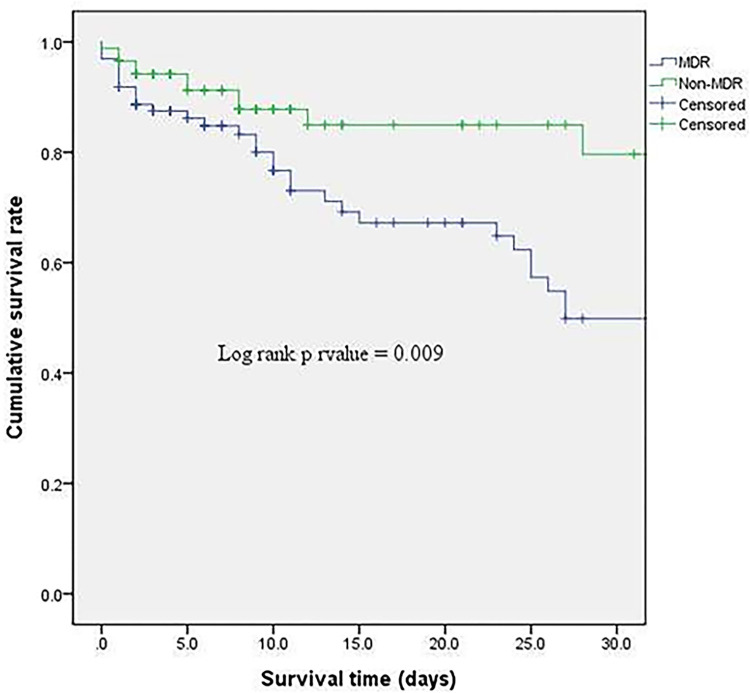
Kaplan–Meier survival curves according to the presence of MDR bacteria.

We also divided and analyzed the CPSS group according to the presence of MDR and their baseline characteristics are shown in **S3 Table**. The MDR group (n = 98 [53.0%]) and the non-MDR group (n = 87 [47.0%]) did not show significant differences in the requirement of MV and RRT, duration of PICU stay, duration of inotropes/vasopressor support and MV. However, the MDR group had a significantly higher 30-day mortality (31.6% vs. 11.6%; *p* = 0.001) and in-hospital mortality (37.8% vs. 20.7%; *p* = 0.015) compared with the non-MDR group (**Table 3**, **Fig 2**).

**Table 3:** Clinical outcomes according to the presence of MDR bacteria.

	MDR (n = 98)	Non-MDR (n = 87)	*p*-value
30-day mortality	31 (31.6)	10 (11.6)	0.001
In hospital mortality	37 (37.8)	18 (20.7)	0.015
Duration of PICU stay (days)	17.6 ± 21.6	19 ± 38.5	0.76
Duration of hospital stay (days)	78.6±105.9	74.2±102.8	0.78
MV requirement	52 (53.1)	42 (48.3)	0.56
RRT requirement	21 (21.4)	14 (16.1)	0.45
Time from recognition of sepsis to antibiotics administration (mins)	38.5 ± 60.9	42.7 ± 85.8	0.70
Duration of vasopressor/inotrope infusion (days)	6.9 ± 7	6.7 ± 7	0.89
Duration of MV (days)	11.7 ± 20.6	8.8 ± 15.3	0.29

Data are n (%) or means ±standard deviation

Abbreviations: CPSS = culture-positive septic shock; CNSS = culture-negative septic shock; PICU = pediatric intensive care unit; MV = mechanical ventilator; RRT = renal replacement therapy

## Discussion

In this study, we found that there was no significant difference in the rate of 30-day mortality and in-hospital mortality between the CPSS group and CNSS group in critically ill pediatric patients. Furthermore, the two groups showed similar results in other clinical outcomes such as the requirement of MV and RRT, duration of PICU stay, and duration of MV/inotropes support. In the CPSS group, those with MDR bacteria had a significantly high 30-day mortality and in-hospital mortality than those without MDR bacteria.

In our study, the proportion of the CNSS group among pediatric patients was not smaller than that of the CPSS group, which is in line with recent retrospective worldwide studies on adult patients [11, 13–15, 23]. There are several reasons for the occurrence of CNSS. First, many patients had already received oral antibiotics at local clinics before obtaining cultures. Also, hospitalized patients are often administered intravenous antibiotics before the onset of sepsis [24, 25]. Second, in the case of immunocompromised patients, viral infection or fungal infection often mimics the clinical features of sepsis [26]. Third, some patients showed clinical features of septic shock from non-infectious diseases, such as inflammatory diseases and hemato-malignancy disease [25, 27, 28].

Studies comparing the clinical features between CPSS and CNSS have shown conflicting results. Appropriate use of antibiotics according to proven pathogen improves the clinical prognosis [6, 29]; therefore, a previous study that reported higher mortality in patients with culture-negative sepsis explained the results as patients with culture-positive sepsis being able to receive appropriate antibiotics by obtaining accurate information about the cause of their sepsis [24]. In contrast, one study found a worse prognosis in patients with culture-positive sepsis and attributed the findings to differences in the patient characteristics such as patient population, distributions of infection sites, and delayed administration of antibiotics [17, 30]. Some studies reported that there was no significant relationship between culture results and clinical prognosis, which is consistent with our results [14, 15, 23]. Those studies also suggested that early administration of appropriate antibiotics and early eradication of bacteria were more important factors than culture results.

Our results on the similar clinical outcomes between the CPSS group and the CNSS group can be explained as follows. First, the two groups had similar PRISM III and pSOFA scores even though the CPSS group had lower systolic blood pressure and lower diastolic blood pressure than the CNSS group. However, because both PRISM III and pSOFA scores consider blood pressure values according to age, it is difficult to say that the difference in absolute values of blood pressure between the two groups is clinically significant. Therefore, the two groups had similar disease severity, which could have contributed to the comparable prognosis. Second, we promptly administered broad-spectrum antibiotics to both groups according to the Surviving Sepsis Campaign guidelines 2020, meaning that we evenly applied appropriate sepsis management and supportive care to the two groups [19]. Furthermore, our patients had routinely undergone culture before starting antimicrobials, which is presented in the rationale of Surviving Sepsis Campaign guidelines 2020 [19].

There are only few studies on the clinical outcomes of pediatric septic shock patients infected with MDR bacteria. However, among the CPSS group, those with MDR bacteria had worse clinical prognoses than those with non-MDR bacteria. However, if bacteria with MDR are detected in the culture, the clinical prognosis would be poor because source control is difficult [31–33]. In our study, patients infected with MDR bacteria, regardless of gram-positivity, had significantly higher short-term and long-term mortality.

The prevalence of underlying disease in our study patients is different from those in previous studies. Specifically, the proportion of hemato-oncology patients is higher than in other studies on adult patients. Patients with hemato-oncology diseases have a different immune function state, which may affect the clinical course of sepsis. As an example, some hemato-oncology patients have neutropenia due to chemotherapy, which may lead to severe immune dysregulation [34]. However, as the proportion of oncology patients was similar between the CPSS group and CNSS group in our study, the underlying disease is not likely to have been a significant factor that influenced the clinical outcomes.

There are potential limitations to our study. This study was a retrospective single-center study that included a relatively small number of pediatric septic shock patients.

Our study has the following strengths. To the best of our knowledge, this is a study to directly compare the clinical features between the CPSS group and CNSS group for pediatric patients. Second, the same management strategy for septic shock was applied in the two groups. Moreover, our study strictly followed the recent sepsis guidelines, such as the timing of administering broad-spectrum antimicrobials and obtaining appropriate routine microbiologic cultures before applying antibiotics. Third, we evaluated not only the clinical characteristics according to the culture results, but also according to antibiotic sensitivity and the subsequent results.

In conclusion, our study showed that there was no significant difference in the clinical features and prognosis between the CPSS group and CNSS group in critically ill pediatric patients. However, among those with CPSS, infection with MDR bacteria was associated with a significantly higher mortality rate.

## Supporting information

S1 Fig The distribution and number of bacteria isolated from culture. (TIF)

S1 Data set (XLSX)

S1 Table Frequency and positivity of culture site. (DOCX)

S2 Table Frequency and mortality according to the bacteria species in the CPSS group. (DOCX)

S3 Table Baseline characteristics according to the presence of MDR bacteria. (DOCX)
