# Supplementary figures and images for: Comparison of the clinical characteristics and clinical outcomes of culture-positive septic shock and culture-negative septic shock among pediatric patients
Source: PLoS One. 2023 Jul 14;18(7):e0288615. doi: 10.1371/journal.pone.0288615 (PMC10348532; doi:10.1371/journal.pone.0288615)

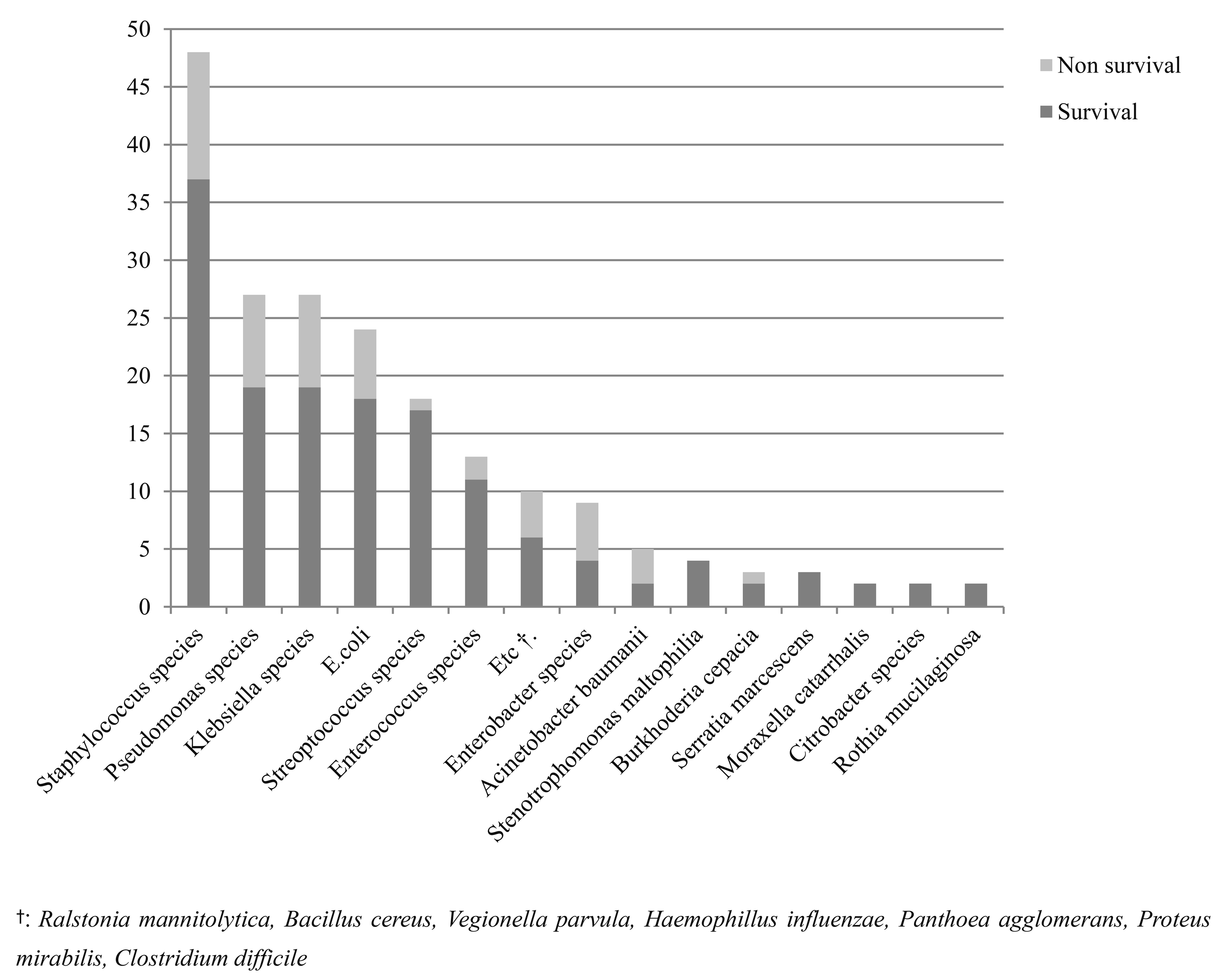

Supplement: S1 Fig — (TIF) [file pone.0288615.s001.tif]
