# Supplementary material for: Comparison of the clinical characteristics and clinical outcomes of culture-positive septic shock and culture-negative septic shock among pediatric patients
Source: PLoS One. 2023 Jul 14;18(7):e0288615. doi: 10.1371/journal.pone.0288615 (PMC10348532; doi:10.1371/journal.pone.0288615)
Supplement: S1 Table — (DOCX) [file pone.0288615.s003.docx]

| Culture site | Frequency | Positivity |
| --- | --- | --- |
| Blood | 289 | 241 (83.4) |
| Sputum | 233 | 71 (30.5) |
| Urine | 165 | 78 (47.3) |
| Stool | 60 | 28 (46.7) |
| Ascites | 19 | 14 (73.7) |
| CSF | 16 | 6 (37.5) |
| Pleural fluid | 13 | 4 (30.8) |
| Other* | 10 | 7 (70) |

*skin wound, ear discharge, joint, nasal discharge, lung tissue, bartholin cyst
