# Supplementary material for: Comparison of the clinical characteristics and clinical outcomes of culture-positive septic shock and culture-negative septic shock among pediatric patients
Source: PLoS One. 2023 Jul 14;18(7):e0288615. doi: 10.1371/journal.pone.0288615 (PMC10348532; doi:10.1371/journal.pone.0288615)
Supplement: S2 Table — (DOCX) [file pone.0288615.s004.docx]

| Species | Frequency, n (%) | Total mortality, n (%) | Net mortality (%) |
| --- | --- | --- | --- |
| Gram-positive |  |  |  |
| *Staphylococcus* species | 48 (25.9) | 11 (16.7) | 22.9 |
| *Streptococcus* species | 18 (9.7) | 1 (1.5) | 5.6 |
| *Enterococcus* species | 13 (7) | 2 (3) | 15.4 |
| Gram-negative |  |  |  |
| *Pseudomonas* species | 27 (14.6) | 8 (12.1) | 29.6 |
| *Klebsiella* species | 27 (14.6) | 8 (12.1) | 29.6 |
| *Escherichia coli* | 24 (13) | 6 (9.1) | 25 |
| *Enterobacter* species | 9 (4.7) | 5 (7.6) | 55.6 |
| *Acinetobacter baumanii* | 5 (2.7) | 3 (4.5) | 60 |
| *Burkhoderia cepacia* | 3 (1.6) | 1 (1.5) | 33.3 |
| *Stenotrophomonas maltophilia* | 4 (2.1) | 0 | 0 |
| *Serratia marcescens* | 3 (1.6) | 0 | 0 |
| *Moraxella catarrhalis* | 2 (1) | 0 | 0 |
| *Citrobacter* species | 2 (1) | 0 | 0 |
| *Rothia mucilaginosa* | 2 (1) | 0 | 0 |
| Other^*^ | 10 (5.4) | 4 (6.1) | 40 |

^*^*Ralstonia mannitolytica, Bacillus cereus, Vegionella parvula, Haemophillus influenzae, Panthoea agglomerans, Proteus mirabilis, Clostridium difficile*
