# Supplementary material for: Comparison of the clinical characteristics and clinical outcomes of culture-positive septic shock and culture-negative septic shock among pediatric patients
Source: PLoS One. 2023 Jul 14;18(7):e0288615. doi: 10.1371/journal.pone.0288615 (PMC10348532; doi:10.1371/journal.pone.0288615)
Supplement: S3 Table — (DOCX) [file pone.0288615.s005.docx]

| Variables | Total (n=185) | MDR (n=98) | Non-MDR (n=87) | *P*-value |
| --- | --- | --- | --- | --- |
| Age (months) | 56.4 (15.5–153.8) | 58.5 (16.7–140.2) | 52.6 (10.8–157.8) | 0.85 |
| Male sex | 115 (62.2) | 60 (61.2) | 55 (63.2) | 0.88 |
| Body weight (kg) | 21.6 ± 18.1 | 21.6 ± 17.7 | 21.7 ± 18.7 | 0.97 |
| Hospital day | 76.5±104.2 | 78.6±105.9 | 74.2±102.8 | 0.78 |
| Underlying disease |  |  |  | 0.36 |
| Cardiac disease | 40 (21.6) | 25 (25.5) | 15 (17.2) |  |
| Pulmonary disease | 38 (20.5) | 19 (19.4) | 19 (21.8) |  |
| Renal disease | 9 (4.9) | 3 (3.1) | 6 (6.9) |  |
| Hemato-oncologic disease | 69 (37.3) | 40 (40.8) | 29 (33.3) |  |
| Liver disease | 8 (4.3) | 5 (5.1) | 3 (3.4) |  |
| Neurologic disease | 12 (6.5) | 1 (1) | 11 (12.6) |  |
| Gastrointestinal disease | 4 (2.2) | 2 (2) | 2 (2.3) |  |
| Metabolic disease | 3 (1.6) | 1 (1) | 2 (2.3) |  |
| No disease | 2 (1.1) | 2 (2) | 0 |  |
| Initial vital signs |  |  |  |  |
| Systolic blood pressure (mmHg) | 73.8 ± 15.6 | 74.5 ± 15.6 | 73 ± 15.6 | 0.50 |
| Diastolic blood pressure (mmHg) | 40.5 ± 11 | 40.3 ± 10.7 | 40.6 ± 11.4 | 0.84 |
| Heart rate (/min) | 150 ±33.2 | 146.3 ± 33.7 | 154.3 ± 32.4 | 0.10 |
| Body temperature (℃) | 37.9 ± 1.3 | 37.8 ± 1.2 | 38 ± 1.4 | 0.33 |
| Medication |  |  |  |  |
| Time to administration of antibiotics (mins) | 40.5 ± 73.5 | 38.5 ± 60.9 | 42.7 ± 85.8 | 0.70 |
| Laboratory |  |  |  |  |
| WBC (×10^3^/uL) | 11.85 ± 13.4 | 10.3 ± 10.8 | 13.6 ± 15.6 | 0.10 |
| Hb (g/dL) | 8.8 ± 1.9 | 8.8 ± 1.8 | 8.8 ± 2 | 0.93 |
| PLT (×10^3^/uL) | 124.6 ± 248.3 | 125 ± 150.4 | 124.2±326.1 | 0.98 |
| PT (INR) | 2.1 ± 2.2 | 2.1 ± 2.3 | 2 ± 2 | 0.79 |
| Lactate (mmol/L) | 5 ± 4.5 | 4.9 ± 4.4 | 5.2 ± 4.6 | 0.69 |
| BUN (mg/dL) | 27.5 ± 21 | 29.2 ± 23.1 | 25.7 ± 18.2 | 0.25 |
| Creatinine (mg/dL) | 1.1 ± 1.5 | 1.1 ± 1.3 | 1.1 ± 1.7 | 0.96 |
| Total bilirubin (mg/dL) | 2.6 ± 5.5 | 3.5 ± 7.3 | 1.7 ± 1.9 | 0.034 |
| CRP (mg/dL) | 16.1 ± 10.9 | 16.4 ± 12 | 15.8 ± 9.5 | 0.71 |
| Albumin (g/dL) | 2.7 ± 0.6 | 2.8 ± 0.7 | 2.7 ± 0.5 | 0.31 |
| Score |  |  |  |  |
| PRISMIII | 13.4 ± 7.8 | 13.8 ± 8.4 | 13 ± 7.1 | 0.48 |
| pSOFA | 9.7 ± 4 | 10.1 ± 3.9 | 9.2 ± 3.9 | 0.11 |

Data are presented as median (interquartile range), n (%), or mean ± standard deviation

Abbreviations: MDR=multidrug resistance; WBC=white blood cell; Hb=hemoglobin; PLT=platelet; PT=prothrombin time; BUN=blood urea nitrogen; CRP=C-reactive protein; PRISM=pediatric risk of mortality; pSOFA=pediatric sequential organ failure assessment
